# The Imaging of Insulinomas Using a Radionuclide-Labelled Molecule of the GLP-1 Analogue Liraglutide: A New Application of Liraglutide

**DOI:** 10.1371/journal.pone.0096833

**Published:** 2014-05-07

**Authors:** Jing Lv, Yu Pan, Xiao Li, Dengfeng Cheng, Shuai Liu, Hongcheng Shi, Yifan Zhang

**Affiliations:** 1 Department of Nuclear Medicine, Ruijin Hospital, School of Medicine, Shanghai Jiao Tong University, Shanghai, China; 2 Department of Nuclear Medicine, Zhongshan Hospital, School of Medicine, Fudan University, Shanghai, China; University of Lancaster, United Kingdom

## Abstract

**Objective:**

This study explores a new, non-invasive imaging method for the specific diagnosis of insulinoma by providing an initial investigation of the use of ^125^I-labelled molecules of the glucagon-like peptide-1 (GLP-1) analogue liraglutide for *in vivo* and *in vitro* small-animal SPECT/CT (single-photon emission computed tomography/computed tomography) imaging of insulinomas.

**Methods:**

Liraglutide was labelled with ^125^I by the Iodogen method. The labelled ^125^I-liraglutide compound and insulinoma cells from the INS-1 cell line were then used for *in vitro* saturation and competitive binding experiments. In addition, in a nude mouse model, the use of ^125^I-liraglutide for the *in vivo* small-animal SPECT/CT imaging of insulinomas and the resulting distribution of radioactivity across various organs were examined.

**Results:**

The labelling of liraglutide with ^125^I was successful, yielding a labelling rate of approximately 95% and a radiochemical purity of greater than 95%. For the binding between ^125^I-liraglutide and the GLP-1 receptor on the surface of INS-1 cells, the equilibrium dissociation constant (K_d_) was 128.8±30.4 nmol/L(N = 3), and the half-inhibition concentration (IC_50_) was 542.4±187.5 nmol/L(N = 3). Small-animal SPECT/CT imaging with ^125^I-liraglutide indicated that the tumour imaging was clearest at 90 min after the ^125^I-liraglutide treatment. An examination of the *in vivo* distribution of radioactivity revealed that at 90 min after the ^125^I-liraglutide treatment, the target/non-target (T/NT) ratio for tumour and muscle tissue was 4.83±1.30(N = 3). Our study suggested that ^125^I-liraglutide was predominantly metabolised and cleared by the liver and kidneys.

**Conclusion:**

The radionuclide ^125^I-liraglutide can be utilised for the specific imaging of insulinomas, representing a new non-invasive approach for the *in vivo* diagnosis of insulinomas.

## Introduction

Insulinoma is the most common type of pancreatic endocrine tumour. Clinically, the inappropriate secretion of insulin by insulinomas often causes recurrent episodes of hypoglycemia. Certain episodes are accompanied by neuropsychiatric manifestations. In severe cases, insulinoma-related symptoms can be fatal.

The best treatment for insulinoma is surgical resection, and accurately locating an insulinoma is critical to successful surgical treatment. Insulinomas, which mainly occur in the pancreas, are usually benign single tumours with diameters of less than 2 cm; these tumours are equally likely to occur in the head, body, or tail of the pancreas [1]. Therefore, it is difficult to accurately locate an insulinoma, particularly in cases involving ectopic insulinomas [2].

Ultrasonography allows for the simple and convenient examination of insulinomas; however, this approach is vulnerable to interference from intestinal gas, abdominal fat, and the spleen. Thus, the overall diagnostic rates with this method are not high; in particular, conventional ultrasonography exhibits a sensitivity of approximately 20%–30% [3]. Although the use of endoscopic ultrasonography can reduce occlusion and interference from gastrointestinal gases and soft tissues in pancreatic examinations, thereby yielding a sensitivity of up to 89% [4], this approach is not only invasive but also produces relatively low diagnostic rates for tumours in the pancreatic tail. It is difficult to detect small insulinomas by conventional computed tomography (CT), whereas pancreatic perfusion CT and dynamic contrast-enhanced magnetic resonance imaging (MRI) can produce improved tumour detection rates [5], [6] but are difficult to utilise for qualitative diagnosis. The sensitivities of conventional CT and MRI for diagnosing insulinoma have been reported to be 55% [7] and 71% [8], respectively. Therefore, further improvement of the sensitivity and specificity of insulinoma diagnoses remains an important issue in the diagnosis of this disease.

The radionuclide-based imaging of somatostatin receptors (SSTRs) has demonstrated high sensitivity and specificity in the diagnosis of neuroendocrine tumours. This approach can be utilised for qualitative diagnoses, accurate staging, and evaluation of treatment efficacy. However, the detection rate of SSTR imaging for insulinoma is not high. Previous research has indicated that the positive imaging rate for the diagnosis of benign insulinoma using ^111^In-labelled octreotide is only 46% [9]. This limited utility is because in tumours, among the five subtypes of SSTRs (SSTR1-SSTR5), radionuclide-labelled octreotide can only bind to SSTR2 and SSTR5 [10]. Moreover, SSTR2 is expressed in only half of insulinomas, which furthermore express SSTRs at a low density [11]. These factors all limit the use of SSTR imaging for locating and diagnosing insulinomas. New technology is being used in the diagnosis of neuroendocrine tumors such as ^68^Ga (DOTATE, DOTANOC and DOTATOC) with PET/CT. Despite the higher spatial resolution could be achieved by using these probes, it presents similar limitiations to scintigraphy with somatostatin analogues in the diagnosis of insulinomas for the same reasons presented. Glucagon-like peptide-1 (GLP-1) is an incretin hormone released from the L cells of the intestine [12] that plays the physiological role of binding to the GLP-1 receptor. The GLP-1 receptor is a G protein-coupled receptor with seven transmembrane domains [13]. The binding of GLP-1 to the GLP-1 receptors of pancreatic β cells contributes to the maintenance of glucose homeostasis primarily by stimulating the release of insulin from these β cells [14]. Because the GLP-1 receptor is overexpressed in insulinomas [15], which have a higher density of GLP-1 receptors than of SSTRs [16], the use of radionuclide-labelled GLP-1 and its analogues for imaging may exhibit greater sensitivity and specificity with respect to locating insulinomas than the labelling of SSTRs. However, exogenous GLP-1 can be rapidly degraded *in vivo* by dipeptidyl peptidase IV (DPP-IV) [17]; as a result, exogenous GLP-1 exhibits a serum half-life of less than 2 min [18] and is therefore unsuitable for radionuclide imaging.

Exendin-4 is a GLP-1 analogue isolated from the saliva of poisonous lizards. The structure of exendin-4 exhibits 53% homology with the structure of GLP-1 [19], and the *in vivo* half-life of exendin-4 is 9.57 h [20]. Exendin-4 can specifically bind to the GLP-1 receptor [19] and is resistant to degradation because it lacks the sites for enzymatic hydrolysis by DPP-IV [21], [22]. In recent years, exendin-4 labelled with the radionuclide ^111^In has been used in clinical imaging to locate insulinomas. In 6 insulinoma patients, insulinomas were successfully detected by GLP-1 receptor imaging, which demonstrated significantly higher sensitivity than conventional CT and MRI [23].

Liraglutide is an acylated GLP-1 analogue in which the lysine at residue 34 of the natural GLP-1 molecule has been replaced by arginine, and the lysine at residue 26 has been modified by the addition of a 16-carbon palmitoyl fatty acid side chain with a glutamic acid spacer [24]. Thus, liraglutide retains the function of natural GLP-1 but is not susceptible to degradation by DPP-IV; in particular, liraglutide has an *in vivo* half-life of up to 13 h [25]. Liraglutide has been widely utilised for the clinical treatment of type 2 diabetes and produced good therapeutic effects. We have previously conducted a study in which ^18^F-labelled exendin-4 was used for the small-animal positron emission tomography (PET)/CT imaging of insulinomas in mice [26]. Liraglutide has 97% homology with GLP-1 [27] and exhibits pharmacological and biological characteristics that are nearly identical to the characteristics of GLP-1. Given these similarities, this study explores a new approach to the specific diagnosis of insulinoma through the first investigation of the use of ^125^I-labelled liraglutide for the small-animal single-photon emission computed tomography (SPECT)/CT imaging of insulinomas.

## Methods and Materials

### Materials

#### **Preparation of ^125^I-liraglutide**

The Iodogen method was used to label liraglutide (purchased from GL Biochem, Shanghai, China) with ^125^I (purchased from Shanghai Xinke, Shanghai, China). In the labelling reaction, 25 µL of liraglutide (2 mg/mL) and 25 µL of Na^125^I in 1× phosphate-buffered saline (PBS) (20 mCi/mL) were sequentially added to a reaction tube coated with Iodogen and mixed gently. The tube was sealed, and the reaction was allowed to proceed for 20 min at room temperature.

After the reaction was completed, the labelling efficiency was determined using a mixture of methanol and water (volume methanol:volume water  = 85∶15) as the developing solvent and Whatman 3 mm chromatography paper as the stationary phase, with Rf values of 0 and 0.8 for ^125^I-liraglutide and Na^125^I, respectively. The final labelling rate of ^125^I-liraglutide was approximately 95%, and the radiochemical purity was greater than 95%. In vitro stability test shows that ^125^I-liraglutide could be kept intact in fetal bovine serum till 8 hours. The stability in fetal bovine serum at 37°C showed 92.9%,92.3%,91.4%,90.6%,88.4% of intact peptide at 0, 2, 4, 6, 8 h, respectively.

### Culturing of INS-1 cells

Cells from the INS-1 insulinoma cell line (purchased from ATCC and provided by the Department of Endocrinology of Ruijin Hospital, which is affiliated with the Shanghai Jiao Tong University School of Medicine,) were cultured in RPMI (Roswell Park Memorial Institute) 1640 medium (Sigma, St. Louis, Missouri, USA) containing 10% foetal bovine serum (FBS) (Hyclone, Logan, Utah, USA), 1% penicillin-streptomycin (Gibco, California, USA), 1% sodium pyruvate (Gibco), 0.1% β-mercaptoethanol (Gibco), and 0.84% HEPES (4-(2-hydroxyethyl)-1-piperazineethanesulfonic acid) (Gibco). The cells were cultured in petri dishes with a diameter of 10 cm (Corning, New York, USA) placed in a cell incubator at 37°C and 5% CO_2_ (Heraeus, Hanau, Germany).

### Receptor saturation binding experiments

The aforementioned Iodogen method was used to label liraglutide with ^125^I, producing a compound with a specific activity of approximately 8.33 µCi/µg.

Well cultured ins-1 cells were digested with 0.25% trypsin (Invitrogen, Carlsbad, California, USA) and centrifuged. The cells were then counted, seeded onto 24-well plates (Corning) to a density of approximately 5×10^5^ cells per well, and cultured in a cell incubator at 37°C and 5% CO_2_. After 24 h, the cells were washed 3 times with 1× PBS. ^125^I-liraglutide was added to the wells until radioactivity levels of 0, 5×10^5^, 1×10^6^, 2×10^6^, 4×10^6^, 8×10^6^, 1×10^7^, 2×10^7^, and 3×10^7^ cpm were reached, and the final volume in each well was adjusted to 500 µl with PBS; thus, the final concentrations of ^125^I-liraglutide in the wells were 0, 14.4, 28.8, 57.6, 115.2, 230.4, 460.8, 921.6, and 1382.4 nmol/L. Three replicate wells were established for each sample. To exclude the effects of non-specific binding, 30 µg of unlabelled liraglutide was added to each well of another plate, with a final solution volume of 500 µl. Again, three replicate wells were established for each sample. The cells in the aforementioned two groups were cultured for 40 min in a cell incubator at 37°C and 5% CO_2_. After incubation, the cells were washed 3 times with cold PBS containing 0.5% bovine serum albumin (BSA). The supernatant was aspirated, and 500 µl of 1 mol/L NaOH was added to each well. After the resulting reaction had proceeded for 5 min, the cells were detached from the bottom of each well by pipetting (eppendorf research of 1000 ul, Germany) and transferred to a tube that was impervious to γ radiation(13×78 mm, Zhejiang Gongdong Medical Plastic Factory, Zhejiang, China). The radioactivity in each tube in counts per minute was measured using a γ counter (Shanghai Rihuan Photoelectric Instrument, Shanghai, China).

The obtained data were processed using the single-site saturation binding curve fitting program from GraphPad Prism 5 (GraphPad Software, La Jolla, California, USA) to calculate the receptor saturation binding curve for ^125^I-liraglutide.The K_d_ and B_max_ values calculated by nonlinear regression analysis using the GraphPad Prism 5.

### Competitive binding experiments

Well cultured iNS-1 cells seeded into 24-well plates at a concentration of approximately 5×10^5^ cells per well, and cultured in a cell incubator at 37°C and 5% CO_2_ for 24 h. Then the cells were washed 3 times with 1× PBS. ^125^I-liraglutide was added to each well to a concentration of 11.2 nmol/L, and unlabelled liraglutide was added in each well to concentrations of 0, 1, 10, 100, 1000, and 10000 nmol/L; the final volume in each well was adjusted to 500 µl with PBS. Three replicate wells were established for each concentration. The aforementioned 24-well plates were incubated in a cell incubator at 37°C and 5% CO_2_ for 40 min. After this incubation, the cells were washed three times with cold PBS containing 0.5% BSA. The supernatant was aspirated, and 500 µl of 1 mol/L NaOH was added to each well. After the resulting reaction was allowed to proceed for 5 min, the cells were detached from the bottom of each well by pipetting (eppendorf research of 1000 ul, Germany)and transferred to a tube that was impervious to γ radiation(13×78 mm, Zhejiang Gongdong Medical Plastic Factory, Zhejiang, China). The radioactivity in each tube in counts per minute was measured using a γ counter.

The obtained data were processed using the single site competitive binding curve fitting program of GraphPad Prism 5 to calculate the IC_50_ value(by nonlinear regression analysis) representing the half-inhibition concentration of liraglutide for ^125^I-liraglutide.

### Establishing a nude mouse model of insulinoma

Female BALB/c nude mice that were 3–4 W of age (purchased from the Experimental Animal Centre of Ruijin Hospital, which is affiliated with Shanghai Jiao Tong University, Shanghai, China) were reared in the Experimental Animal Centre of Ruijin Hospital, which is affiliated with Shanghai Jiao Tong University, under specific pathogen-free (SPF) conditions. All animal experiments were approved by the Ethics Committee of the Shanghai Jiao Tong University School of Medicine and were conducted in accordance with ethical principles governing animal welfare, rearing, and experimentation.

A total of 24 nude mice were each subcutaneously injected in the armpit of the right upper limb with 100 µl of a suspension of INS-1 cells (containing approximately 10^7^ cells per 100 µl). The nude mice were reared under conventional sterile conditions and given 5% dextrose injections as drinking water (Shanghai Changzheng Fumin Jinshan Pharmaceutical, Shanghai, China). Their blood glucose levels were monitored every 2–3 days with a glucometer (Accu-Chek Integra integrated blood glucose meter, Roche, Berlin, Germany), and all measured blood glucose levels were at least 3 mmol/L.

After approximately four weeks of rearing, the tumours grew to 0.5–1.0 cm, and the mice were used for small-animal SPECT/CT imaging.

### Small-animal SPECT/CT imaging for the nude mouse model of insulinoma

In this experiment, 100 µl of ^125^I-liraglutide solution, which contained approximately 800 µCi of ^125^I-liraglutide, was injected into the tail veins of each of 3 nude mice with insulinoma. Each nude mouse was placed into an induction chamber prior to imaging, and the mice were anesthetised with 5% isoflurane. At 30 min, 90 min, 180 min, and 360 min after the injection, the nude mice were placed on a small animal SPECT/CT scanning bed (Trifoil, formerly Bioscan, Washington, USA). During the image acquisition period, the nude mice were anesthetised with 1–2% isoflurane delivered through a custom face mask. CT images were acquired (Number of Projections:180; Pitch:1; Tube Voltage:55 kev; Exposure Time:1000 ms; Binning:1∶4), followed by Small-animal SPECT images(Scan mode:Standard; Frame time: 30 s; Frame Size:256×256; Energy peak:28.0 kev; Full Width:20%)

### Blocking the small-animal SPECT/CT imaging for the nude mouse model of insulinoma

In this experiment, 100 µl of liraglutide solution containing 300 µg of unlabelled liraglutide was injected into the tail veins of each of 3 nude mice with insulinoma. Half an hour after this injection, 100 µl of ^125^I-liraglutide solution, which contained approximately 800 µCi of ^125^I-liraglutide, was injected into the tail veins of each of these mice. To create an experimental group that corresponded to this blocked group, 100 µl of ^125^I-liraglutide solution containing approximately 800 µCi of ^125^I-liraglutide was injected into the tail veins of each of 3 more nude mice with insulinoma. Prior to imaging, the nude mice were placed into an induction chamber and anesthetised with 5% isoflurane. At 90 min after the ^125^I-liraglutide injection, the nude mice were fixed onto a small-animal SPECT/CT scanning bed. During the image acquisition period, the nude mice were again anesthetised with 1–2% isoflurane delivered through a custom face mask. CT images were acquired (Number of Projections:180; Pitch:1; Tube Voltage:55 kev; Exposure Time:1000 ms; Binning:1∶4), followed by Small-animal SPECT images(Scan mode:Standard; Frame time: 30 s; Frame Size:256×256; Energy peak:28.0 kev; Full Width:20%)

### The processing of small-animal SPECT/CT images

The data collected from the small-animal SPECT/CT scans were reconstructed using the Nuclear v1.02 software package. The images were collected using the HiSPECT 1.4.2 software package, and analysed using InVivoScope 1.44 software. Here are the reconstruction parameters of CT (In-Plane Voxel Size:Medium; Slice Thickness: Medium; Filter: Butterworth, Cut Off:100%; Voxel size: 146 um×146 um×146 um) and the reconstruction parameters of SPECT (OSEM Algorithm; Iteration 9 Subset 20)

### The *in vivo* distribution of ^125^I-liraglutide radioactivity across various organs

Six nude mice each received a 100 µl injection containing approximately 100 µCi of ^125^I-liraglutide. At 90 min and 360 min after this injection, 3 mice were sacrificed by cervical dislocation. First, blood was collected from the heart; next, isolated various organs and tissues, including the heart, lungs, liver, stomach, kidneys, spleen, small intestine, and pancreas, as well as muscle tissue and the tumour; the isolated samples were then placed into tubes that were impervious to γ radiation and weighed. For each tube, the radioactivity in counts per minute was then measured by a γ counter. The results were presented in terms of %ID/g (the percentage of the total injected dose of radioactivity per gram of tissue). Histograms of %ID/g for various organs at the 90 min and 360 min time points were plotted.

### *In vivo* blocking experiment

In this experiment, 100 µl of liraglutide solution containing 100 µg of unlabelled liraglutide, was injected into the tail veins of each of 3 nude mice with insulinoma. Half an hour later, 100 µl of ^125^I-liraglutide solution containing approximately 100 µCi of ^125^I-liraglutide was injected into the tail vein of each mouse. To create an unblocked experimental group that corresponded with this blocked group, 100 µl of ^125^I-liraglutide solution containing approximately 100 µCi of ^125^I-liraglutide was injected into the tail veins of each of 3 more nude mice with insulinoma. These six nude mice with insulinoma were sacrificed by cervical dislocation at 90 min after the ^125^I-liraglutide injection. First, blood was collected from the heart and various organs and tissues were isolated; the isolated samples were then placed into tubes that were impervious to γ radiation and weighed. For each tube, the radioactivity in counts per minute was then measured by a γ counter. The results were presented in terms of %ID/g. A histogram of %ID/g for various organs at the 90 min time point in the blocked and unblocked groups was plotted.

### Statistical analysis

Data are presented as the mean ± SD of at least N = 3, and statistical analysis was performed using Student's t-test. A p value of less than 0.05 was considered statistically significant.

## Results

### Saturation curve

**Figure 1:**
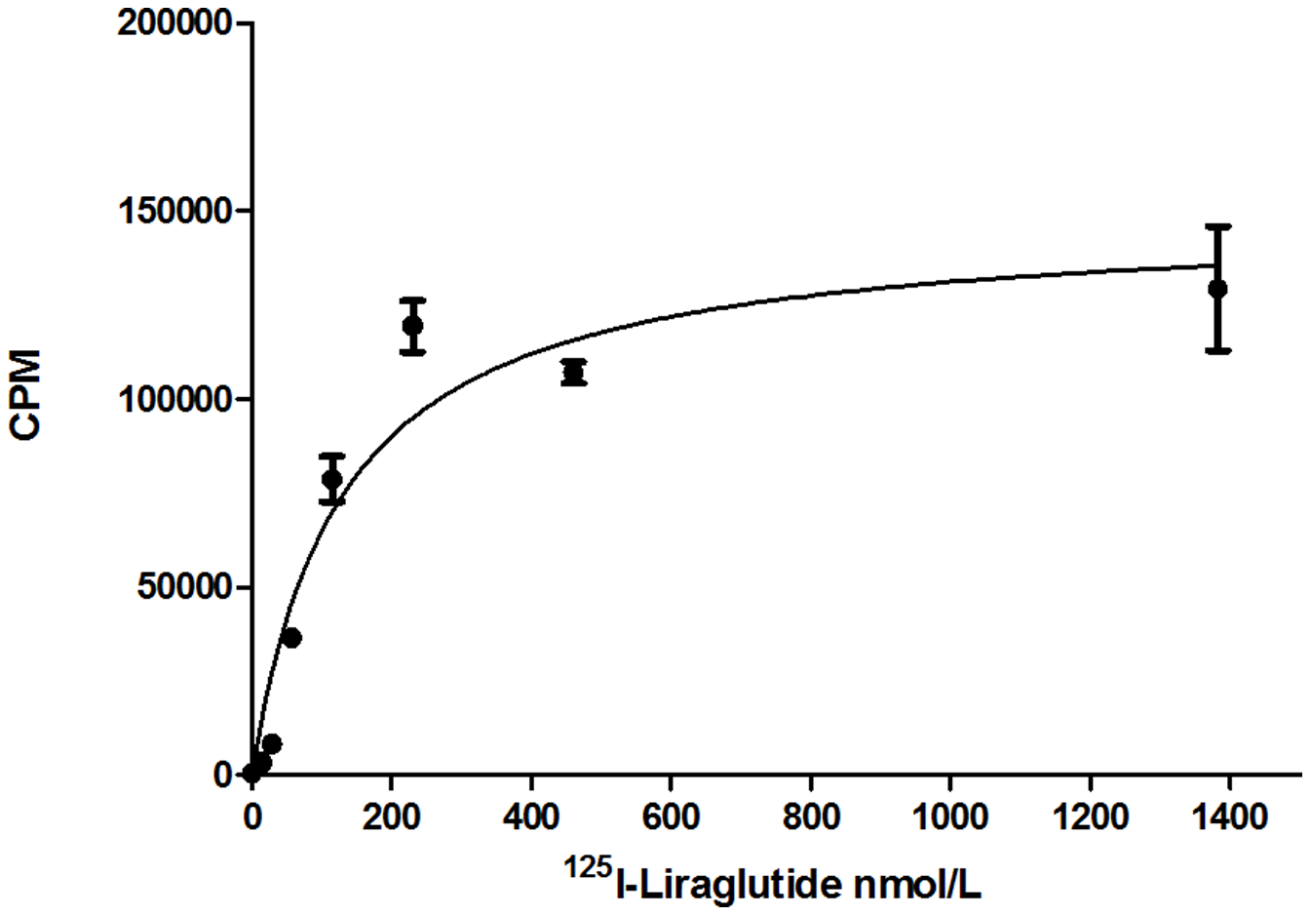
Saturation curve. The equilibrium dissociation constant K_d_ for the binding between ^125^I-liraglutide and the GLP-1 receptors of INS-1 cells was 128.8±30.4 nmol/L, and the maximum binding constant B_max_ was (1.50±0.23)×10^5^. Error bars represent 1 SD of the mean(N = 3).

Graphpad Prism 5 was used to fit the results of the *in vitro* binding experiment to a saturation curve (Figure 1), which eventually approaches saturation. The equilibrium dissociation constant K_d_ for binding between ^125^I-liraglutide and the GLP-1 receptor of INS-1 cells was calculated to be 128.8±30.4 nmol/L(N = 3), and the maximum binding constant B_max_ was determined to be ^(^1.50±0.23) ×10^5^ (N = 3).

### Competition binding curve

**Figure 2:**
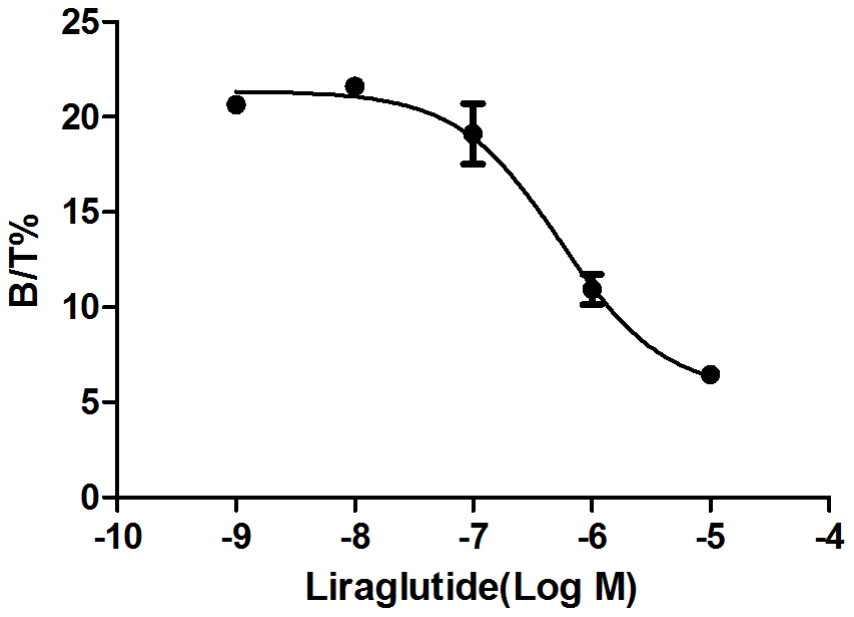
Competitive binding curve. IC_50_, the half-inhibition concentration of liraglutide, was 542.4±187.5 nmol/L. Error bars represent 1 SD of the mean(N = 3).

Because the GLP-1 receptor is highly expressed by INS-1 cells [28], a competitive binding curve could be fit using Graphpad Prism 5 to describe the binding of liraglutide and ^125^I-liraglutide to this receptor (Figure 2). The IC_50_ value representing the half-inhibition concentration for the inhibition of ^125^I-liraglutide by liraglutide was calculated to be 542.4±187.5 nmol/L(N = 3).

### Small-animal SPECT/CT imaging with ^125^I-liraglutide in the nude mouse model

**Figure 3:**
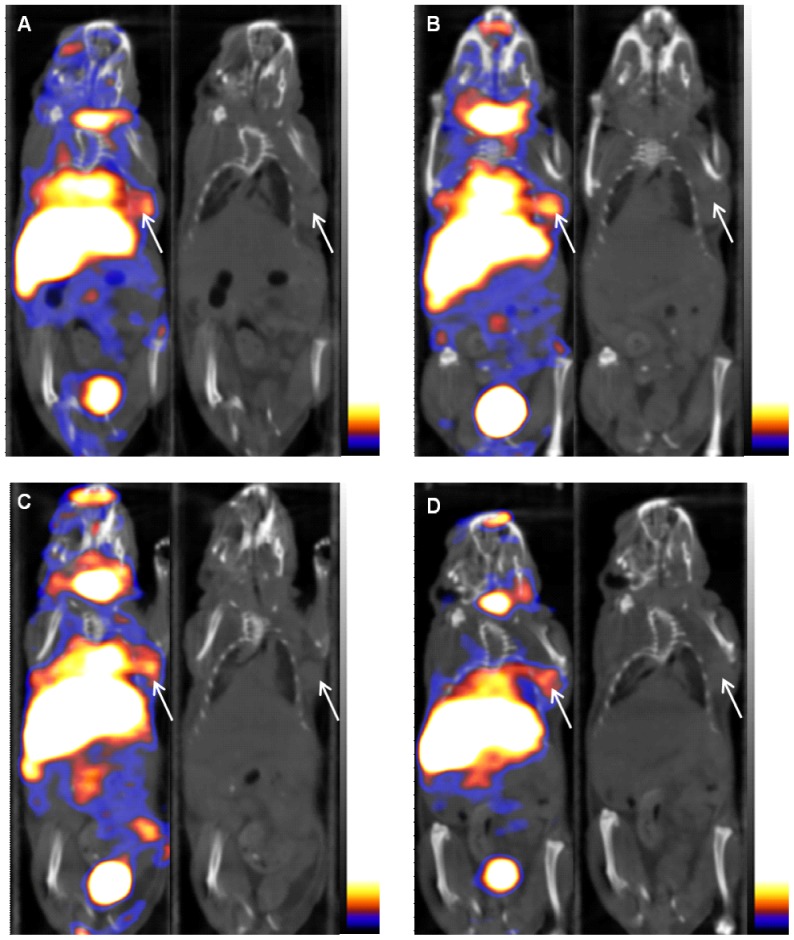
Small-animal SPECT/CT imaging in the insulinoma model. Small-animal SPECT/CT imaging after the injection of ^125^I-liraglutide into the tail veins of nude mice indicated that the tumour could be clearly imaged at 30 min after this injection (**A**), with the clearest images of the tumour obtained at 90 min after the injection (**B**). The tumour had begun to fade by 180 min after the injection (**C**) and had significantly faded by 360 min after the injection (**D**). In addition, the thyroid, liver, heart, lung, kidney, bladder, and other organs were visible to various degrees in the imaging results. The CT coronal tomographic images correspond to the SPECT/CT images, and the arrow indicates the tumour location.

Small-animal SPECT/CT imaging using the established insulinoma model (Figure 3 A, B, C, D) clearly revealed the tumour at 30 min after the injection of ^125^I-liraglutide into the tail vein of a mouse with insulinoma; the tumour was clearest at 90 min after this injection, but the tumour image had begun to fade by 180 min after the injection and had significantly faded by 360 min after the injection. In addition, images of the thyroid, liver, heart, lungs, kidneys, bladder, and other organs were visible to various degrees in the images, although radioactivity remained at low levels in the muscle tissue throughout the experiment. The coronal CT images in Figure 3 correspond to the SPECT/CT imaging results for nude mice, with the arrows indicating the xenograft tumour in the armpit area.

Due to the small size of the pancreas in nude mice and the resolution limitations of small-animal CT, CT imaging cannot be used to visualise the pancreas. In small-animal SPECT/CT, sites corresponding to the pancreas exhibited no significant uptake of radioactivity, but imaging clearly revealed tumours, with significant contrasts between the appearance of the tumours and the appearance of the pancreatic region.

### Determinations of the radioactivity distribution across various organs in the nude mice model

**Figure 4:**
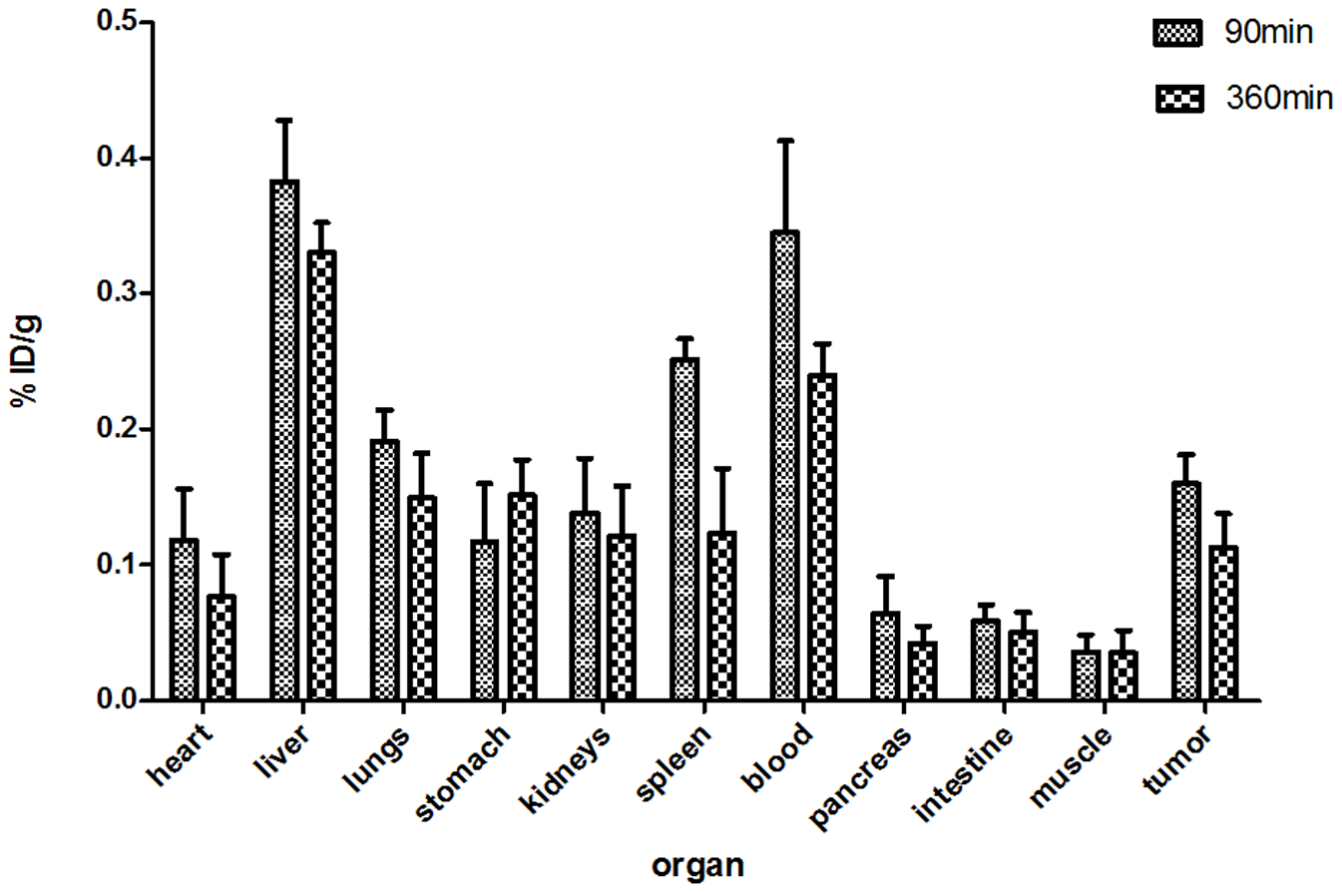
Histograms of the %ID/g for various organs of nude mice at 90 min and 360 min after an injection of ^125^I-liraglutide. At 90%ID/g ± SD(N = 3).

Histograms of %ID/g for various organs at 90 min and 360 min after an injection of ^125^I-liraglutide (Figure 4) revealed that at 90 min, radioactivity was highest in the liver and blood, followed by the spleen, lungs, tumour, kidneys, heart, and stomach, with relatively low radioactivity in the pancreas and small intestine. By 360 min, radioactivity had decreased to varying degrees in all organs except the stomach. Minimal levels of radioactivity were observed in the muscle tissue at 90 min after an injection of ^125^I-liraglutide, with no significant change in radioactivity at 360 min. Radioactivity in the stomach increased from 90 min after the injection of ^125^I-liraglutide to 360 min after this injection. At 90 min, the tumour tissue exhibited a high distribution of radioactivity, with even higher levels of radioactivity in the tumour than in the kidneys and the heart. The T/NT ratios for the muscle and pancreatic tissues were4.83±1.30(N = 3) and2.73±0.80(N = 3), respectively.

### The blocking of small-animal SPECT/CT imaging with 125I-liraglutide in the nude mouse model

**Figure 5:**
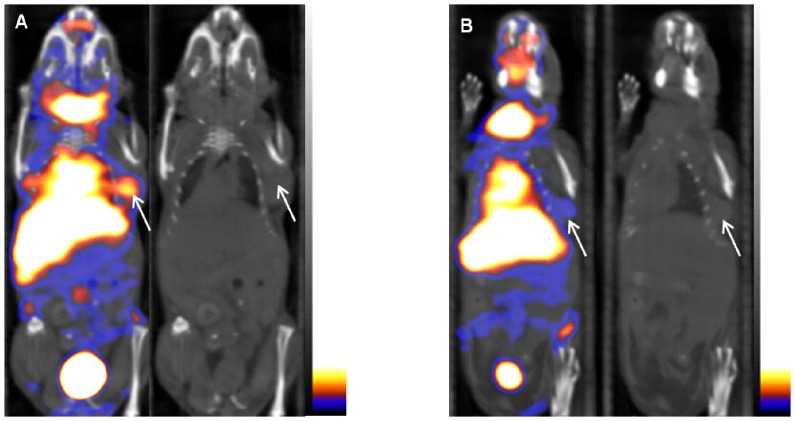
Imaging of the nude mouse model of insulinoma 90 min after blocking with liraglutide. Small-animal SPECT/CT imaging indicated that tumour images significantly faded after blocking with liraglutide (**B**), while **A** presents an image obtained prior to blocking. The images of the lungs also faded after blocking. The coronal CT images correspond to the SPECT/CT images, and the arrow indicates the tumour location.

**Figure 6:**
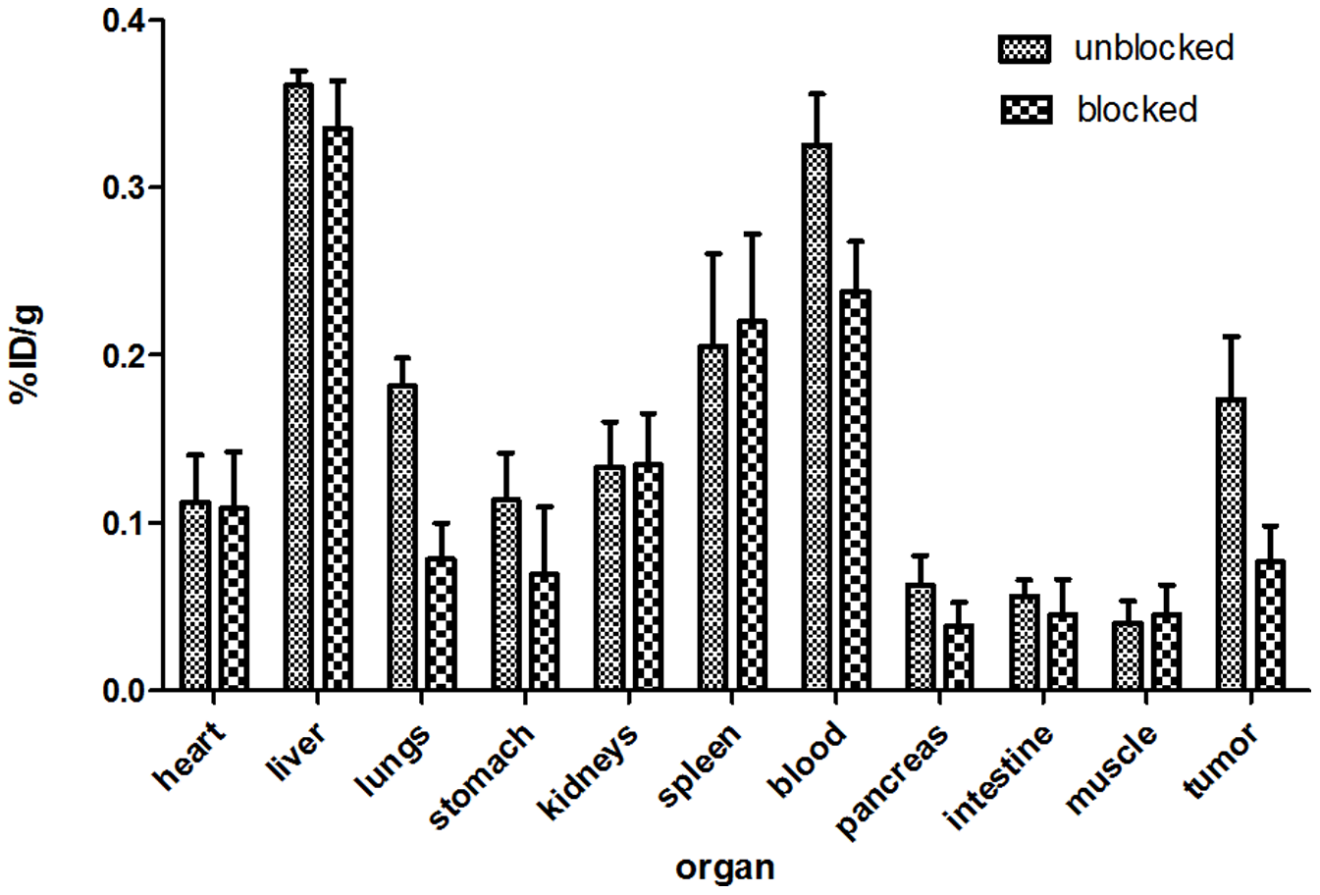
A histogram of %ID/g for various organs in a model mouse 90 min after blocking with liraglutide. The radioactivity of the tumour(p<0.05) was significantly reduced after blocking, and blocking caused the radioactivity to decrease to varying degrees in the lungs(p<0.05), stomach, pancreas(p<0.05), and other organs. Student t test for significance, p<0.05(statiscally significant p values are marked in brackets). Data are expressed as average %ID/g ± SD(N = 3).

Small-animal SPECT/CT imaging with ^125^I-liraglutide at 90 min revealed that after blocking with liraglutide, the radioactivity at the tumour site was significantly reduced, and the lung image had faded (Figure 5B). Figure 5A presents images obtained prior to blocking. The coronal CT images in Figure 5A and Figure 5B correspond to the SPECT/CT images. Histograms of the distribution of radioactivity across different organs before and after blocking (Figure 6) indicated that blocking significantly reduced the radioactivity of the tumour(p<0.05) and produced varying degrees of decreases in radioactivity in the lungs(p<0.05), stomach, pancreas(p<0.05), and blood. Blocking caused no significant changes in radioactivity in the other examined organs.

## Discussion

Liraglutide is already used for the clinical treatment of diabetes in Europe, the United States, and other countries. Liraglutide treatment can continuously improve haemoglobin A1c (HbA1c) levels, reduce body weight, and improve the function of islet β cells in patients with type 2 diabetes [24]. Because of liraglutide's long *in vivo* half-life, the once-daily administration of liraglutide can achieve good hypoglycemic effects [25], and severe hypoglycemic reactions rarely occur. Thus, liraglutide is currently the most widely recognised GLP-1 receptor agonist [29].

We previously conducted a study of small-animal PET/CT imaging of insulinomas using ^18^F-labelled exendin-4 [26]. Based on this prior study, we considered examining liraglutide for insulinoma imaging because liraglutide has 97% homology with natural GLP-1 [27] and exhibits virtually all of the biological characteristics of GLP-1 and because the GLP-1 receptor is highly expressed in insulinomas [15]. Therefore, in this study, we conducted the first investigation of the use of liraglutide labelled with the radionuclide iodine-125 (^125^I) for *in vivo* imaging. Because the structure of liraglutide includes tyrosine groups, direct iodine labelling only minimally impacts the *in vivo* distribution of this polypeptide. Thus, the labelled polypeptide can fairly directly reflect the biological characteristics of liraglutide and may be preliminarily assessed. Moreover, there are convenient sources of ^125^I, and ^125^I is simple to use for labelling. ^125^I,with a half-life of almost 60 days, emits a very low average energy of 28.5 kev by electron capture (EC). These factors are directly related to the radiation dose exposed to individual and the lower quality of image compared to other iodine radioisotopes such as ^123^I, ^124^I, and ^131^I. Although ^125^I is not routinely used as a radionuclide in clinical SPECT imaging, ^125^I has the same chemical characteristics as ^123^I, ^124^I, and ^131^I, and the same labelling methods may be utilised for any of these iodine isotopes. The biological characteristics of the label will remain unchanged regardless of which isotope is used; thus, similar imaging results should be obtained with each isotope.

The *in vitro* saturation binding experiment indicated that the binding of ^125^I-liraglutide to the GLP-1 receptors of INS-1 cells was saturable and specific, with a maximum binding constant B_max_ of up to ^(^1.50±0.23)×10^5^(N = 3) and an equilibrium dissociation constant K_d_ of 128.8±30.4 nmol/L(N = 3). The competitive binding experiment revealed that the half-inhibition concentration IC_50_ of liraglutide was 542.4±187.5 nmol/L(N = 3), indicating that ^125^I-liraglutide exhibited a high affinity for binding to GLP-1 receptors. However, compared with the K_d_ (56.64 nmol/L) and IC_50_(0.35–2.81 nmol/L) in the study of exendin-4 imaging we formly conducted, the affinity of liraglutide with GLP-1 receptors seems lower despite two different insulinoma cell lines(INS-1 and Rin-m5f).

*In vivo* small-animal ^125^I-liraglutide SPECT/CT imaging using the established insulinoma model indicated that the tumour could be imaged at 30 min after an injection of ^125^I-liraglutide and that the imaging results for the tumour were clearest at 90 min after this injection but had faded by 180 min after the injection. The liver, blood, lungs, kidneys, bladder, and other organs were clearly visible in the images. In a preliminary experiment, it was observed that the imaging results for tumours were clearer at 60 minutes after an injection of ^125^I-liraglutide than at 30 min; the tumours became clearest at 90 min after the injection but had begun to fade by 120 min after the injection. Therefore, the four time points of 30 min, 90 min, 180 min, and 360 min were selected for imaging procedures using the nude mouse model established in this study.

The distribution of %ID/g of ^125^I-liraglutide for various organs of the examined nude mice also indicated that 90 min after an injection of ^125^I-liraglutide, radioactivity was highest in the liver and blood, followed by the spleen, lungs, and kidneys. Because liraglutide has a large molecular weight of approximately 3751 daltons, it is primarily metabolised by the liver and cleared by the kidneys; this trait is consistent with the general metabolic patterns for polypeptides. Because liraglutide was injected at high doses and has a long *in vivo* half-life (approximately 13 h), high levels of radioactivity were found in the blood, and the heart image was evident. Because GLP-1 receptors are highly distributed on the surface of mouse lung cells [30], the imaging clearly revealed the lungs of the examined mice. In this study, there was significantly lower radioactivity in the myocardial tissues of the heart than in the blood. The distribution of radioactivity in the muscle tissues remained at low levels throughout the experiments. The *in vivo* metabolism of liraglutide led to reductions in the radioactivity in relevant organs. However, the radioactivity in the stomach was greater at 360 min after an injection of ^125^I-liraglutide than at 90 min after the injection; this observation may relate to the increased *in vivo* deiodination of ^125^I-liraglutide over time, which could lead to the uptake of free I^−^ by the blood of the gastric mucosa. Similarly, the thyroid could be imaged due to the uptake of free iodine ions from the bloodstream.

Insulinomas are mostly located in the pancreas, and SPECT/CT imaging in the nude mouse model revealed no significant uptake of radioactivity at inferred pancreatic sites, while the uptake of radioactivity was evident in the tumour at the implantation site. The *in vivo* distribution of radioactivity at 90 min after an injection of ^125^I-liraglutide showed that the T/NT ratio for tumour and muscle tissues was4.83±1.30(N = 3), and the T/NT ratio for tumour and pancreatic tissue was 2.73±0.80(N = 3); accordingly, insulinomas are clearly evident in the SPECT/CT imaging results. In addition, the high radioactivity exhibited to various extents by the liver, spleen, stomach, and kidneys could affect the use of planar SPECT imaging for the diagnosis of pancreatic tumours; however, tomography may be utilised in SPECT/CT imaging to avoid interference from nearby organs in examinations of the pancreas.

The *in vivo* imaging experiments involving blocking revealed that the blocking caused significant fading in the imaging results for both the tumours and the lungs. After blocking, the *in vivo* ID%/g distribution across various organs also indicated that radioactivity had significantly decreased in the lungs, tumour and pancreas. This decrease occurred because the aforementioned tissues and organshave GLP-1 receptors. Liraglutide blocked the binding of ^125^I-labelled liraglutide to the GLP-1 receptors of these tissues and organs. This finding indicated that the biological characteristics of ^125^I-labelled liraglutide were the same as the biological characteristics of unlabelled liraglutide and that ^125^I-labelled liraglutide binds specifically to the GLP-1 receptor. The reduction in radioactivity in the blood associated with blocking could relate to the dilution of the ^125^I-liraglutide in the bloodstream by liraglutide, the injected blocking agent. The ID%/g of the stomach was also reduced after blocking; this reduction may have occurred because GLP-1 receptors also exist in gastric tissue, as reported by Gotthardt *et al.* [30]. No blocking in other organs was found in the *in vivo* study; these findings were consistent with the *in vivo* distribution of GLP-1 receptors across bodily organs.

Gotthardt *et al.* [30] conducted a prior study in which the GLP-1 analogue exendin-3 was labelled with the radionuclide ^123^I and utilised for the *in vivo* imaging of RIN-m5F insulinomas in rats. The resulting findings demonstrated that ^123^I-exendin-3 could specifically bind to insulinomas for imaging, suggesting that radionuclide imaging of the GLP-1 receptor was a novel method for diagnosing insulinomas. However, exendin-3 does not contain a tyrosine group; therefore, radioactive iodine was used to label a histidine group, with poor labelling effectiveness. Wild *et al.* [31] used exendin-4 instead of exendin-3; specifically, exendin-4 was labelled with the radionuclide ^111^In to create {[Lys^40^ (Ahx-DTPA-^111^In)NH_2_] Exendin-4)}, which was used for the imaging of insulinomas in transgenic Rip1Tag2 mice. The results of these experiments indicated that the labelled exendin-4 could specifically bind to insulinomas, further supporting the idea that GLP-1 receptor imaging is an effective method for locating insulinomas.

The diagnosis and location of insulinomas typically involves selective pancreatic angiography, transhepatic portal venous sampling (THPVS), endoscopic ultrasound, and/or other invasive procedures. Although these approaches exhibit high sensitivity, they are all invasive examinations, and their clinical application involves certain risks. However, non-invasive examination techniques, such as CT, MRI, and ultrasound, are not highly specific and continue to encounter particular difficulties with the diagnosis of insulinomas of less than 2 cm [32].

Currently, imaging with radionuclide-labelled octreotide is the primary method for the functional imaging of insulinomas; however, this approach is associated with a positive imaging rate of only 46% [9]. Given the much greater structural homology between liraglutide and GLP-1 (97%) than between exendin-4 and GLP-1 (53%), the use of liraglutide for diagnosing insulinoma could produce improved diagnostic sensitivity and specificity compared to the current approaches. This novel approach to the clinical location and diagnosis of insulinoma merits exploration. Because GLP-1 receptors also exist in islet β cells [33], we speculate that liraglutide might also provide new imaging solutions for monitoring the distribution and survival of islet cells after transplantation.

This study involved the first examination of liraglutide labelling with the radionuclide ^125^I. Because ^125^I cannot be used for clinical diagnosis, to further the development of clinical applications, the labelling of liraglutide with the radionuclide ^99m^Tc for SPECT imaging may be attempted. In addition, the spatial resolution of gamma camera is approximately 0.8 to 1 cm and insulinomas are less than 2 cm in diameter mostly, therefore,its sensitivity for detection can be hampered by the resolution of the method. Thus, the labelling of liraglutide with positronic ^18^F or other positronic radionuclides could be used in PET imaging to improve diagnostic sensitivity.

## Conclusions

^125^I-labelled liraglutide was used as a new radiotracer that can bind to insulinoma cells with high affinity and specificity. Small-animal SPECT/CT imaging using this radiotracer clearly depicted tumours; thus, this approach provides a new method for the specific imaging of insulinomas. In future studies, we will select more clinically suitable radionuclide labels for SPECT or PET imaging to study and develop the potential clinical value of liraglutide-based imaging techniques.
